# Poor Outcomes of Children and Adolescents with Femoral Neck Fractures: A Meta‐Analysis Based on Clinical Studies

**DOI:** 10.1111/os.12629

**Published:** 2020-03-11

**Authors:** Yingpu Chen, Xiaojun Zhang, Hao Guo, Na Liu, Jing Ren, Chao Lu

**Affiliations:** ^1^ Department of Orthopedics Xi'an Hong Hui Hospital, Xi'an Jiaotong University Health Science Center Xi'an China; ^2^ Department of Orthopedics People's Hospital of Tongchuan Tongchuan China

**Keywords:** Avascular necrosis, Closed reduction, Femoral neck fracture, Meta‐analysis, Open reduction

## Abstract

**Objective:**

To comprehensively assess the differences in outcome between open reduction and closed reduction for children and adolescents with femoral neck fractures.

**Methods:**

Based on the predetermined strategies, eligible studies were obtained by searching Embase, the Cochrane Library, and PubMed databases (retrieval time: June 2018) and through manual retrieval for paper documents. The 95% confidence intervals (CI) and risk ratios (RR) were used as evaluation indexes. Moreover, the results of avascular necrosis, coxa vara, or non‐union were compared between open reduction and closed reduction under random or fixed effects models. After sensitivity analysis was carried out, publication bias was evaluated for the eligible studies using Egger's test.

**Results:**

Six studies were included in our meta‐analysis. No significant heterogeneity was found among the included studies (*P* ≥ 0.05) and, thus, the fixed effects model was used for merging the effect sizes of avascular necrosis (RR [95% CI] = 0.50 [0.26, 0.98], *P* = 0.04), coxa vara (RR [95% CI] = 0.16 [0.04, 0.70], *P* = 0.01), and non‐union (RR [95% CI] = 0.22 [0.05, 0.93], *P* = 0.04). Sensitivity analysis suggested that the results of avascular necrosis were not stable (RR = 0.50, 95% CI = 0.25 1.17, *P* = 0.12), while those of coxa vara and non‐union were stable. There was no significant publication bias among the eligible studies (*t* = −0.70, *P* = 0.522).

**Conclusion:**

Femoral neck fractures treated by open reduction had less adverse outcomes compared with those treated by closed reduction.

## Introduction

Femoral fractures are induced by serious trauma and include fractures of the neck, the head, the trochanter, the middle of the femur, and the diaphysis^1^. Femoral fractures are often characterized by severe pain, swelling, leg shortening, deformity, soft‐tissue injury, shock, and bleeding^2^, ^3^, ^4^. Avascular necrosis of the femoral head and nonunion of fractures are the two major complications in the therapy of femoral neck fractures^5^. The optimal treatment method of femoral neck fractures is manual reduction and internal fixation, and the healing rate is 80% to 90%^6^. Therefore, the treatment principle of femoral neck fractures is early non‐traumatic reduction, reasonable multiple nail fixation, and early recovery^7^.

The reduction quality and the displacement degree of the fracture are related to the prognosis of avascular necrosis following internal fixation of femoral neck fractures^8^. Avascular necrosis is an adverse outcome of femoral neck fractures, and adequate reduction and internal fixation may reduce avascular necrosis after osteosynthesis^9^. The femoral head avascular necrosis rate in the elderly can be decreased through closed reduction and fixation, and reduction quality, fracture type, and the timing of the operation are closely correlated with femoral head avascular necrosis^10^. Several studies have explored the risk differences of avascular necrosis in femoral neck fractures in children and adolescents who have received open reduction or closed reduction. For example, percutaneous cannulated screw fixation and closed reduction are effective in treating adolescents with femoral neck fractures, and minimal time from trauma to operation, rigid internal fixation, and good reduction can reduce the incidence of avascular necrosis and complications^11^. Through comparing the treatment effects of closed reduction and open reduction for femoral neck fractures, closed reduction is found to have lower complication rates (including nonunion fracture healing and avascular necrosis)^12^, ^13^. However, the results of different studies are not consistent. For femoral neck fractures, the therapeutic efficacies and postoperative complications of open reduction and internal fixation (ORIF) and closed reduction and internal fixation (CRIF) are similar and have no significant differences^14^. A meta‐analysis by Wang et al (2014) evaluated the correlation between avascular necrosis and open/closed reduction for femoral neck fractures, revealing that CRIF leads to a higher avascular necrosis rate compared with ORIF^12^. However, that meta‐analysis was not focused on femoral neck fractures in children and adolescents. Therefore, it is necessary to identify the differences in outcomes of children and adolescents treated for femoral neck fractures with open reduction and closed reduction through a more comprehensive meta‐analysis.

Thus, in this study, we use a meta‐analysis to comprehensively assess the differences in outcome between open reduction and closed reduction for children and adolescents with femoral neck fractures. The present study may provide direction for further correlation studies.

## Methods

### *Search Strategy*

Using “femoral neck fractures” and “open reduction” as search key words, eligible studies were extracted from Embase, the Cochrane Library, and PubMed databases based on the predetermined strategies (retrieval time 7 June 2018, without restriction on the language). Combining topic words and free words, the search was performed. The search procedure of PubMed is shown in Supplementary Table S1. Manual retrieval for paper documents was also carried out, and the references of relevant reviews and enrolled studies were further screened to include more studies into this meta‐analysis.

### *Inclusion Criteria*

Studies were considered for inclusion if they met the following criteria.

(i) Participants: Children and adolescents (<18 years old) who suffered from femoral neck fractures.

(ii) Interventions and comparisons: The study explored the differences in poor outcomes between open reduction and closed reduction.

(iii) Outcomes: Avascular necrosis, coxa vara, or non‐union.

(iv) Study design: The study was a randomized controlled trial (RCT) or a non‐randomized clinical study.

### *Exclusion Criteria*

The exclusion criteria were: (i) the studies were reviews, comments, letters, et al; and (ii) the studies were republished papers or studies using data involved in several publications.

### *Data Extraction*

Two investigators independently completed document screening according to the above inclusion and exclusion criteria. After determining the studies to include, the investigators independently performed data extraction in accordance with the predesigned standardized form. The information that needed to be extracted included: the name of the first author, the publication year, the study area, the ages and sexes of the subjects, the sample sizes, the study types, and the research outcomes. After the above data extraction was finished, the two investigators exchanged the extraction tables. If there were any inconsistencies, they discussed these together to resolve disagreements.

### *Statistical Analysis*

The 95% confidence intervals (CI) and risk ratios (RR) were selected as the evaluation indexes. Cochran's *Q* test and the *I* ^2^ test were used for performing heterogeneity tests for the included studies. When the studies had significant heterogeneity (*P* < 0.05 and/or *I* ^2^ > 50%), the random effects model was used for merging the effect sizes. When there were homogeneous outcomes (*P* ≥ 0.05 and *I* ^2^ ≤ 50%), the fixed effects model was used. Egger's test was used to evaluate whether there was publication bias among the included studies. If significant publication bias was found, the effect of publication bias on the results would be assessed with the trim and fill method^15^. Using the transform merge model, a sensitivity analysis was conducted to evaluate the stability of the results. All statistical analysis was performed using RevMan 5.3 software and STATA 11.0 software.

## Results

### *Eligible Studies*

**Figure 1:**
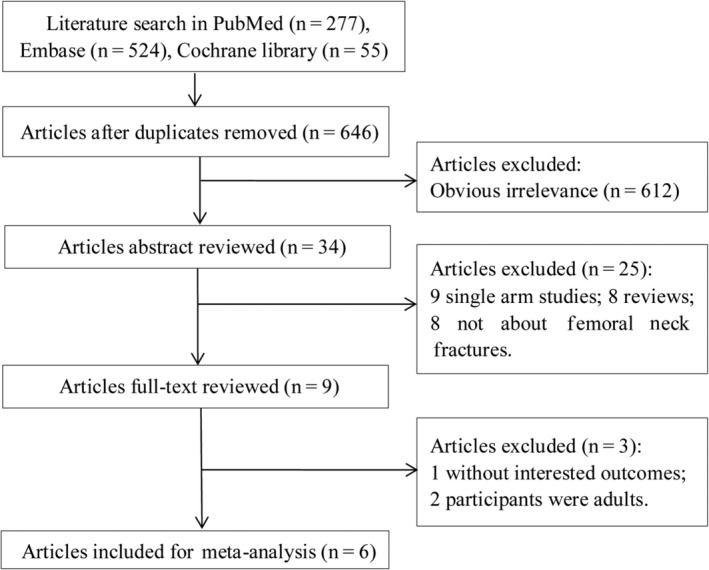
The process of selecting eligible studies. A total of 856 studies were included in this meta‐analysis through a database search; 210 studies were excluded by screening the repeated articles and 612 ineligible studies were removed after scanning through the titles. From the remaining 34 studies, 25 studies were screened out after reading abstracts and 3 studies were further eliminated after reading full texts. Finally, a total of 6 studies with 198 patients were included in this meta‐analysis.

As shown in Fig. 1, a total of 277, 524, and 55 studies were selected from PubMed, Embase, and Cochrane Library databases. Repeated articles were filtered out, and 646 studies remained. Then, 612 ineligible studies were removed after scanning through the titles. From the remaining 34 studies, 25 studies were screened out following reading the abstracts and 3 studies were further eliminated after reading full texts. Finally, a total of 6 studies were included in this meta‐analysis^13^, ^16^, ^17^, ^18^, ^19^, ^20^. As shown in Table 1, there were 198 patients in these 6 studies^13^, ^16^, ^17^, ^18^, ^19^, ^20^, including 118 males and 80 females. The fracture type of the study by Bali *et al*. included 16 type II, 11 type III, and 9 type IV^16^. The fracture type of the study by Dendane *et al*. included 9 type II, 10 type III, and 2 type IV^17^. The fracture type of the study by Ju *et al*. included 30 type II, 21 type III, and 7 type IV^13^. The fracture type the study by Lin *et al*. included 25 type II and 9 type III^18^. The fracture type of the study by Song included 15 type II and 12 type III^19^. The fracture type of the study by Stone *et al*. included 13 type II, 8 type III, and 1 type IV^20^.

**Table 1:** Characteristics of the studies included in the meta‐analysis

Study	Area	Duration	n, M/F	Age, years*	Fracture type (Delbet's criteria)	Displaced fractures, n (Y/N)	Time (days)†	Follow‐up, years	Design	Assessment of outcomes
Bali (2011)	India	1998.05–2007.12	36, 20/16	10 (3–16)	16 type II, 11 type III, and 9 type IV	28/8	NR	2.7 (1.1–9.5)*	RS	Ratliff criteria
Dendane (2010)	Maroc	1999–2006	21, 14/7	12.1 (5–16)	9 type II, 10 type III, and 2 type IV	21/0	0.5–21	1.1	RS	Ratliff criteria
Ju (2016)	China	2005.05–2014.05	58, 40/18	9.1 (1.7–15.6)	30 type II, 21 type III, and 7 type IV	58/0	4.7 (2–12)	2.9 (1.4–5.1)	RS	Ratliff criteria
Lin (2012)	China	2006.03–2010.05	34, 20/14	<16	25 type II, 9 type III	34/0	NR	1.1 (1–3)	RS	Ratliff criteria
Song (2010)	Korea	1989.02–2007.03	27, 11/16	<16	15 type II, 12 type III	27/0	24 cases<1	2.8 (1.3–13)	RS	Ratliff criteria
Stone (2015)	USA	2003–2012	22, 13/9	11.5 (4.5–17.3)	13 type II, 8 type III, and 1 type IV	22/0	18 cases ≤1, 4 cases >1	2.1 (1–8.7)	RS	Ratliff criteria

* Mean (range)

† Between the occurrence of the injury and surgery

F, female; M, male; N, no; NR, not report; RS, retrospective study; Y, yes.

### *Study Characteristics*

All of the 6 included studies were retrospective clinical studies^13^, ^16^, ^17^, ^18^, ^19^, ^20^. The publication years of the included studies were from 2010 to 2016, and their study areas included China^13^, ^18^, India^16^, Korea^19^, the USA^20^ and Morocco^17^. Except for the study of Bali *et al*. (involving 28 displaced fracture cases and 8 undisplaced fracture cases)^16^, the studies only included displaced fracture cases^13^, ^17^, ^18^, ^19^, ^20^. The characteristics and the outcomes of the eligible studies are presented in Tables 1 and 2, respectively.

**Table 2:** Outcomes of the studies included in the meta‐analysis

Study	Area	Group	n, M/F	Fracture type (Delbet's criteria)	Age, years	Delay (days)	AVN	Coxa vara	Non‐union
Bali, 2011	India	ORIF	18, NR	NR	NR	NR	3	0	0
		CRIF	13, NR	NR	NR	NR	3	1	2
Dendane, 2010	Maroc	ORIF	13, 8/5	5 type II, 6 type III, 2 type IV	9–16	1–21	5	NR	NR
		CRIF	8, 6/2	4 type II, 4 type III, 0 type IV	5–14	0.5–15	1	NR	NR
Ju, 2016	China	ORIF	37, 26/11	19 type II, 14 type III, 4 type IV	9.21 ± 3.14	4.89 ± 2.47	5	0	0
		CRIF	21, 14/7	11 type II, 7 type III, 3 type IV	8.85 ± 3.79	4.29 ± 2.10	6	3	1
Lin, 2012	China	ORIF	19, 11/8	14 type II, 5 type III	8.1 ± 1.3	7.3 ± 2.6 hours	0	0	NR
		CRIF	15, 9/6	11 type II, 4 type III	7.9 ± 1.5	8.5 ± 1.8 hours	2	1	NR
Song, 2010	Korea	ORIF	15	8 type II, 7 type III	9.7 (5.0–15.0)	12 cases <1	0	0	0
		CRIF	12	7 type II, 5 type III	10.1 (5.0–16.0)	12 cases <1	2	2	2
Stone, 2015	USA	ORIF	6, 6/0	2 type II, 4 type III, 0 type IV	12.2 (10.7–12.6)	3 cases ≤1, 3 cases >1	0	NR	0
		CRIF	16, 7/9	11 type II, 4 type III, 1 type IV	10.7 (9.3–14)	15 cases ≤1, 1 cases >1	8	NR	2

AVN, avascular necrosis; CRIF, closed reduction and internal fixation; F, female; M, male; NR, not report; ORIF, open reduction and internal fixation.

### *Meta‐analysis*

**Figure 2:**
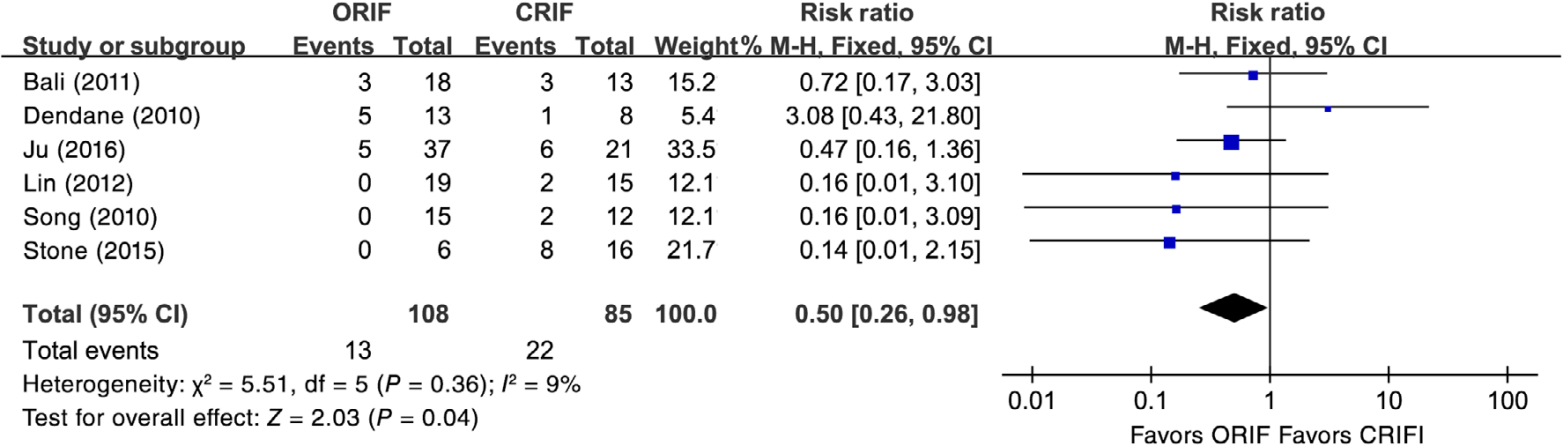
The results of the meta‐analysis for avascular necrosis for open reduction and internal fixation (ORIF) and closed reduction and internal fixation (CRIF). The results of the meta‐analysis for avascular necrosis showed that there was no significant heterogeneity among the 6 included studies (*I* ^2^ = 9%, *P* = 0.36), and the data were pooled using fixed effects model analysis. *P* < 0.05, difference was statistically significant.

**Figure 3:**
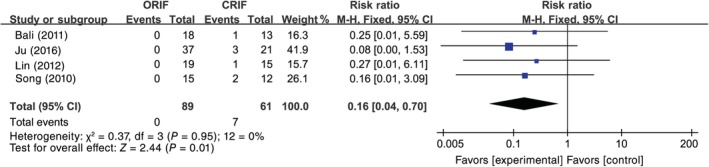
The results of the meta‐analysis for coxa vara between open reduction and internal fixation (ORIF) and closed reduction and internal fixation (CRIF). The meta‐analysis for coxa vara suggested that there were homogeneous outcomes among four eligible studies (*I* ^2^ = 0%, *P* = 0.95), and the data were pooled using fixed effects model analysis. *P* < 0.05, difference was statistically significant.

**Figure 4:**
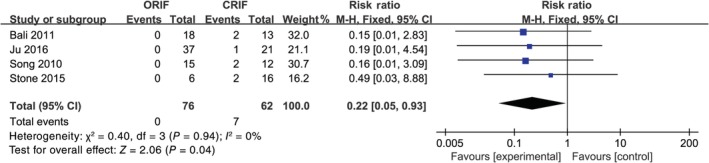
The results of the meta‐analysis for non‐union between open reduction and internal fixation (ORIF) and closed reduction and internal fixation (CRIF). The meta‐analysis results of non‐union indicated that there was no significant heterogeneity in 4 studies (*I* ^2^ = 0%, *P* = 0.94), and the data were pooled using fixed effects model analysis. *P* < 0.05, difference was statistically significant.

The results of the meta‐analysis for avascular necrosis showed that there was no significant heterogeneity among the 6 included studies^13^, ^16^, ^17^, ^18^, ^19^, ^20^ (*I* ^2^ = 9%, *P* = 0.36), and, thus, the fixed effects model was used (RR [95% CI] = 0.50 [0.26, 0.98], *P* = 0.04) (Fig. 2). The meta‐analysis for coxa vara suggested that there were homogeneous outcomes among 4 eligible studies (*I* ^2^ = 0%, *P* = 0.95)^13^, ^16^, ^18^, ^19^, and the fixed effects model was selected (RR [95% CI] = 0.16 [0.04, 0.70], *P* = 0.01) (Fig. 3). Moreover, the meta‐analysis results for non‐union indicated that there was no significant heterogeneity in 4 studies (*I* ^2^ = 0%, *P* = 0.94)^13^, ^16^, ^19^, ^20^, and the fixed effects model was also used (RR [95% CI] = 0.22 [0.05, 0.93], *P* = 0.04) (Fig. 4).

### *Sensitivity Analysis and Publication Bias*

Due to the low heterogeneity among the included studies, the fixed effects model was used for merging the effect sizes of avascular necrosis, coxa vara, and non‐union. The merged results for avascular necrosis changed significantly (RR = 0.50, 95% CI = 0.25 1.17, *P* = 0.12), indicating that the results were not stable. Although the combined results of coxa vara and non‐union were changed, they were still statistically significant. Therefore, the results for coxa vara and non‐union were stable (Table 3).

**Table 3:** Random‐effects and fixed‐effects models comparing open reduction to closed reduction

Outcomes	n	RR (95%CI), *P* value		Test of heterogeneity
		Fixed‐effects model	Random‐effects model	
AVN	6	0.50 (0.26, 0.95), 0.04	0.54 (0.25, 1.17), 0.12	*I* ^2^ = 9%, *P* = 0.36
Coxa vara	4	0.16 (0.04, 0.70), 0.01	0.17 (0.04, 0.76), 0.02	*I* ^2^ = 0%, *P* = 0.95
Non‐union	4	0.22 (0.05, 0.93), 0.04	0.22 (0.05, 0.98), 0.049	*I* ^2^ = 0%, *P* = 0.94

AVN, avascular necrosis; n, number of included trials.

All 6 eligible studies had the outcome of avascular necrosis and, thus, publication bias was analyzed based on avascular necrosis. Egger's test showed that there was no significant publication bias among the included studies (*t* = −0.70, *P* = 0.522).

## Discussion

A total of 6 studies were included into the present meta‐analysis, all of which were retrospective clinical studies. The meta‐analysis for all avascular necrosis, coxa vara, and non‐union showed no significant heterogeneity among the included studies, and, thus, the fixed effects model was used for the three outcomes. The sensitivity analysis showed that the results for avascular necrosis were not stable, while the results for coxa vara and non‐union were stable. Egger's test suggested that there was no significant publication bias among the eligible studies.

Bali *et al*. analyzed the outcomes of femur neck fractures in children treated by CRIF, by ORIF, or conservatively, and found that children treated by ORIF have the lowest rate of complications^16^. Ju *et al*. found that ORIF can generate better outcomes for children with displaced femoral neck fractures compared to closed reduction, which may be correlated with the higher reduction quality of open reduction^13^. Lin *et al*. compared the effects of early anatomical ORIF and CRIF for children with femoral neck fractures and revealed that early ORIF can achieve better reduction and induce fewer complications^18^. ORIF provides a higher quality of reduction and fewer complications than CRIF; therefore, ORIF is a better reduction method^19^. Stone *et al*. report that children with fully displaced femoral neck fractures treated by ORIF had fewer complications (including osteonecrosis) and better reduction in comparison to those treated by CRIF^20^. However, Dendane *et al*. demonstrate that open reduction, late surgery, and older age may increase the occurrence of complications in femoral neck fracture and serve as predictors of the development of avascular necrosis^17^. These inconsistent findings of the 6 included studies might be a result of the different study areas and sample sizes. Thus, this meta‐analysis was critical for quantitatively evaluating the diversity of the 6 eligible studies.

This study is the first meta‐analysis to comprehensively investigate the differences in adverse outcomes of open reduction and closed reduction in treating children and adolescents with femoral neck fractures. There was no significant heterogeneity among the included studies, and most of the demographic and clinical information, such as age and fracture types, for the two groups was not statistically different. Moreover, there was no significant publication bias among the included studies. Despite the above advantages, several limitations also existed in the current study. Only a few studies were included and the sample size was small. Sensitivity analysis showed that the stability of the merged results for avascular necrosis was poor, and the small sample size might be one of the reasons. The results of Dendane *et al*.^17^ were RR (95% CI) = 3.08 (0.43, 21.80). The weight increased from 5.4% to 14.2% under the random effect model, which also led to a significant change in the merger results. Because the included studies were retrospective clinical studies and there was no suitable tool for quality evaluation, this meta‐analysis did not conduct a quality evaluation for the included studies. Therefore, these findings need to be supported by more researches.

In conclusion, the risk of adverse outcomes of open reduction was lower compared with closed reduction. However, more rigorous and high‐quality RCT with large sample sizes are needed to confirm our results.

## Supporting information


**Table S1** The search procedure of PubMed.
