# Supplementary material for: Poor Outcomes of Children and Adolescents with Femoral Neck Fractures: A Meta‐Analysis Based on Clinical Studies
Source: Orthop Surg. 2020 Mar 11;12(2):639–44. doi: 10.1111/os.12629 (PMC7189036; doi:10.1111/os.12629)
Supplement: Supplementary file 1 — Table S1 The search procedure of PubMed. [file OS-12-639-s001.docx]

Supplementary Table 1 The search procedure of PubMed.

| Search | Query | Items found |
| --- | --- | --- |
| #1 | ("femoral neck fractures"[MeSH Terms] OR ("femoral"[All Fields] AND "neck"[All Fields] AND "fractures"[All Fields]) OR "femoral neck fractures"[All Fields]) | [13022](https://www.ncbi.nlm.nih.gov/pubmed/?cmd=HistorySearch&querykey=43) |
| #2 | ("open fracture reduction"[MeSH Terms] OR ("open"[All Fields] AND "fracture"[All Fields] AND "reduction"[All Fields]) OR "open fracture reduction"[All Fields] OR ("open"[All Fields] AND "reduction"[All Fields]) OR "open reduction"[All Fields]) | [40075](https://www.ncbi.nlm.nih.gov/pubmed/?cmd=HistorySearch&querykey=44) |
| #3 | #1 AND #2 | [277](https://www.ncbi.nlm.nih.gov/pubmed/?cmd=HistorySearch&querykey=45) |
